# The RING ubiquitin E3 RNF114 interacts with A20 and modulates NF-*κ*B activity and T-cell activation

**DOI:** 10.1038/cddis.2014.366

**Published:** 2014-08-28

**Authors:** M S Rodriguez, I Egaña, F Lopitz-Otsoa, F Aillet, M P Lopez-Mato, A Dorronroso, S Lobato-Gil, J D Sutherland, R Barrio, C Trigueros, V Lang

**Affiliations:** 1 Cancer Unit, Inbiomed, San Sebastian, Gipuzkoa, Spain; 2 CIC bioGUNE, Derio, Bizkaia, Spain; 3 Cytometry and Advanced Optical Microscopy Core Facility, Inbiomed, San Sebastian, Gipuzkoa, Spain; 4 Hematological Diseases, Inbiomed, San Sebastian, Gipuzkoa, Spain

## Abstract

Accurate regulation of nuclear factor-*κ*B (NF-*κ*B) activity is crucial to prevent a variety of disorders including immune and inflammatory diseases. Active NF-*κ*B promotes I*κ*B*α* and A20 expression, important negative regulatory molecules that control the NF-*κ*B response. In this study, using two-hybrid screening we identify the RING-type zinc-finger protein 114 (RNF114) as an A20-interacting factor. RNF114 interacts with A20 in T cells and modulates A20 ubiquitylation. RNF114 acts as negative regulator of NF-*κ*B-dependent transcription, not only by stabilizing the A20 protein but also I*κ*B*α*. Importantly, we demonstrate that in T cells, the effect of RNF114 is linked to the modulation of T-cell activation and apoptosis but is independent of cell cycle regulation. Altogether, our data indicate that RNF114 is a new partner of A2O involved in the regulation of NF-*κ*B activity that contributes to the control of signaling pathways modulating T cell-mediated immune response.

Nuclear factor-*κ*B (NF-*κ*B) is a principal transcriptional regulator playing a pivotal part in innate and adaptive immunity, inflammation, development, cell proliferation and survival.^1, 2^ Defects in the regulation of NF-*κ*B-dependent gene expression contribute to a variety of diseases, including inflammatory and autoimmune diseases, neurological disorders and cancer.^3, 4, 5^ Therefore, activation of NF-*κ*B is tightly regulated by several NF-*κ*B target genes such as I*κ*B*α*, A20 and CYLD that function as inhibitors in a negative feedback loop.^6, 7, 8, 9, 10^ A20 (also known as TNFAIP3) is a cytoplasmic zinc-finger protein that was originally identified as a tumor necrosis factor (TNF)-inducible protein and it has been characterized as a dual inhibitor of NF-*κ*B activation and cell death.^11^ In most cell types, basal A20 expression is very low but its transcription is rapidly induced upon NF-*κ*B activation. The essential role of A20 in the regulation of NF-*κ*B and apoptotic signaling was clearly demonstrated with the generation of a complete A20 knockout mouse.^12, 13^ Mice deficient for A20 are hypersensitive to TNF and die prematurely because of severe multiorgan inflammation and cachexia.^12^ However, the anti-apoptotic function of A20 is not a general feature, as A20 only protects some cell types from specific death-inducing agents.^14^ Protein ubiquitylation plays an important role in the regulation of NF-*κ*B pathway, not only by controlling the stability of factors integrating this signaling cascade but also their activity. Little is known about the molecular mechanisms that regulate ubiquitin-editing and NF-*κ*B-inhibitory function of A20. Up to date, two enzymatic activities have been associated to A20, a C-terminal ubiquitin ligase and a N-terminal de-ubiquitylating activity, acting on targets such as RIP and promoting their degradation.^15^ A number of A20 interacting proteins including TAX1BP1, Itch and RING-type zinc-finger protein 111 (RNF11) are known to be required for A20 to terminate NF-*κ*B signaling.^16, 17, 18^ Interestingly, the expression, biological activities and mechanism of action of A20 are likely dependent on the cellular context as well as the stimulus involved.^14^ Indeed, in lymphoid cells, A20 is constitutively expressed and its expression is reduced because of activation of the paracaspase MALT1 after T-cell receptor (TCR) stimulation as well as to proteasome degradation.^19^ In addition, in mesenchymal stromal cells, we have recently demonstrated that A20 is constitutively expressed and its expression is reduced after TNF*α* stimulation because of its proteasome-induced degradation.^20^

In humans, polymorphisms within the A20 genomic region predispose individuals to autoimmune diseases such as systemic lupus erythematous, Crohn's disease and psoriasis.^21^ To identify new psoriasis susceptibility loci, a genome-wide association study (GWAS) of 1409 psoriasis patients and 1436 controls was carried out.^22^ Next to single-nucleotide polymorphisms (SNPs) in genes involved in IL-23 signaling, loci including A20, ABIN-1 (also known as TNFAIP3-interacting protein 1 (TNIP1)) and RNF114 showed strong association with psoriasis.^22, 23^

RNF114 belongs to a recently defined family of RING (really interesting new gene) domain E3 ubiquitin ligases, characterized by the presence of three zinc-fingers and one ubiquitin interacting motif (UIM).^24, 25^ RNF114, also known as zinc-finger protein 313 (ZNF313), efficiently binds K48- and K63-linked polyubiquitin chains *in vitro* and *in vivo* and possess an E3 ubiquitin ligase activity. RNF114 is a soluble cytosolic protein that can be induced by interferons and synthetic dsRNA. Real-time PCR analysis demonstrated that RNF114 is clearly expressed in disease-relevant cell types, including CD4+ T lymphocytes, dendritic cells and skin, and also in testis, pancreas, kidney and spleen, indicating that the activity of the RNF114 protein is unlikely to be restricted to the immune system.^26, 27^ Recently, it was observed that RNF114 has a mitogenic function and that its deregulation can disturb cell cycle control mechanism and thus influence cellular stress response. RNF114 expression is reduced at the G1 phase but increased at the S and G2/M transition, suggesting that its elevation may drive a G1 to S transition of the cell cycle.^28^ Using a two-hybrid approach we found that RNF114 was able to interact with A20. Therefore, the goal of this work was to determine the role of this interaction on the stability and activity of A20 and to explore its impact on the regulation of NF-*κ*B-dependent functions.

## Results

### RNF114 interacts with A20

**Figure 1:**
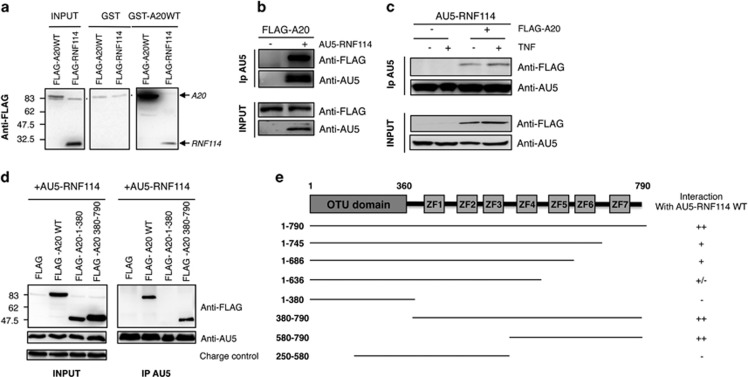
RNF114/ZNF313 interacts with A20. (**a**) Pull-down experiment using GST-A20 or GST fusion proteins and lysates of HEK293 cells transfected with FLAG-A20WT or FLAG-RNF114 is shown (* indicates unspecific band). (**b**) HEK293 cells were transfected with FLAG-A20WT and when indicated with AU5-RNF114. AU5-RNF114 immunoprecipitation was used to confirm the interaction with FLAG-A20. (**c**) HEK293 cells were transfected with AU5-RNF114 and FLAG-A20WT as indicated. Cells were treated with TNF*α* for 20 min and lysates were submitted to anti-AU5 immunoprecipitation (**d**) HEK293 cells were transfected with different forms of FLAG-A20 (WT, N-terminal: 1–390, C-terminal: 390–790) and AU5-RNF114 to determine which domains were involved in the interaction between A20 and RNF114. (**e**) Different constructs of A20 were prepared to define its interaction domain with RNF114. Results of immunoprecipitation experiments are shown. The symbol ‘−' indicates no interaction and ‘+' indicates interaction

To find new A20 interacting proteins, a yeast two-hybrid screening was performed using human thymocytes (CD4+ CD8+) cDNA library and a full-length form of A20 (Hybrigenics, Paris, France). In this screening, three of the proteins found, A20 itself, ABIN-1 and 14-3-3, were already described as A20 interacting proteins.^29, 30, 31^ The RING finger protein RNF114 was identified as a novel interacting protein. This interaction was confirmed using different approaches. First, a pull-down experiment using GST-A20 or GST fusion proteins and lysates of human embryonic kidney 293 (HEK293) cells overexpressing FLAG-RNF114 or FLAG-A20 (Figure 1a) was performed as well as co-immunoprecipitations assays using HEK293 cells transfected with FLAG-A20WT in the presence or not of AU5-RNF114 (Figure 1b). The immunoprecipitation experiment with anti-AU5 antibody showed a clear interaction between FLAG-A20WT and AU5-RNF114 only when both proteins were present. Signal was never detected in the immunoprecipitation control, indicating that this interaction was specific (Figure 1b). Those controls were included only in the first figure to simplify the rest of the figures. TNF*α* stimulation stabilizes FLAG-A20WT, favoring its interaction with AU5-RNF114 (Figure 1c).

To define which part of A20 was involved in its interaction with RNF114, different constructs of A20 were made. In the first experiment, we observed that the C-terminal part of A20 (390–790), containing the E3 ligase domain, was involved in its interaction with RNF114 (Figures 1d and e). To better define the domain of interaction, truncated forms of the C-terminal part were made. Altogether, the results shown in the Figure 1e demonstrate that zinc-fingers 4, 5, 6 and 7 of A20 are contributing to create a solid interaction with RNF114.

**Figure 2:**
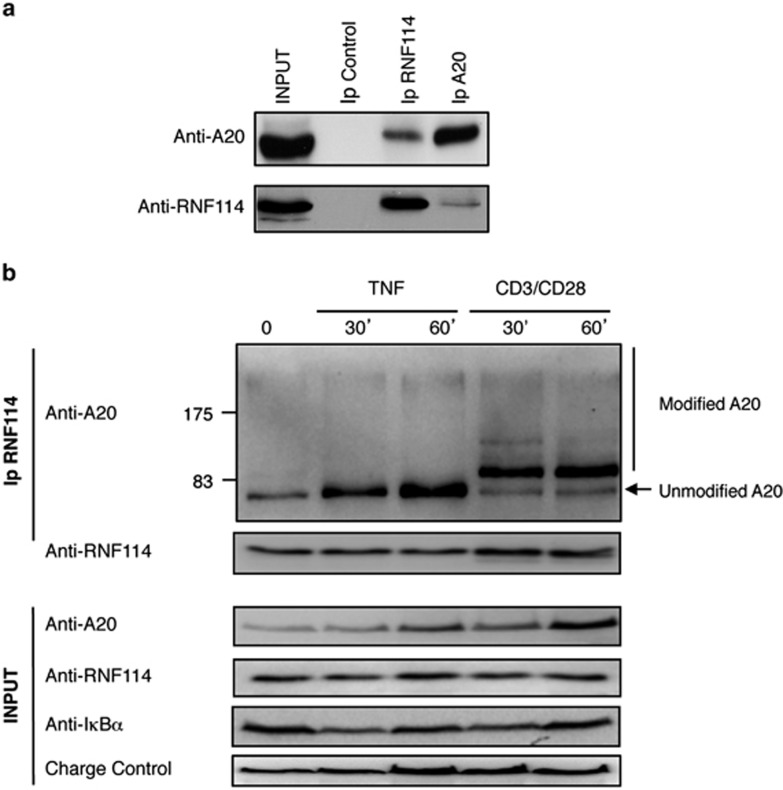
Interaction between endogenous RNF114 and A20 in T cells. (**a**) Jurkat T cells were used to confirm the endogenous interaction between the two proteins. Co-immunoprecipitations of A20 and RNF114, using anti-A20 and anti-RNF114 antibodies, are shown. (**b**) Co-immunoprecipitation between A20 and RNF114 in Jurkat T cells stimulated with TNF*α* or CD3/CD28 for the indicated times using anti-RNF114 antibodies

Finally, to further confirm the association between the two proteins, we checked their interaction at the endogenous level in the absence of any exogenous expression. As A20 is expressed at basal conditions in T cells, we decided to evaluate the interaction between these two proteins in Jurkat T cells by doing a co-immunoprecipitation experiment using anti-A20- or anti-RNF114-specific antibodies. We confirmed the association between these two proteins in reciprocal experiments, even if the interaction was more obvious when the anti-RNF114 antibody was used to co-immunoprecipitate A20 (Figure 2a). This result suggests that only a fraction of A20 is associated to RNF114. However, we cannot exclude that those differences reflect the capacity of each antibody to recognize and bind these interacting molecules (Figure 2a). We checked whether the interaction between these two proteins was modified after stimulation. For that purpose, Jurkat T cells were stimulated as indicated with TNF*α* or CD3/CD28 antibodies. We observed that the association increased after TNF*α* stimulation (Figure 2b), likely as a consequence of an increase in A20 levels after such stimuli. Interestingly, after TCR stimulation, we observed an increase in A20-RNF114 interaction and also a striking modification of A20 molecular weight associated with RNF114 (Figure 2b). These results indicate that under these stimulation conditions, the fraction of A20 able to interact with RNF114 was post-translationally modified. This modified form of A20 is not detectable in the whole lysate extract (INPUT) or after A20 immunoprecipitation (data not shown), supporting the notion that this modified form of A20 specifically bound to RNF114 is a small fraction of the total A20 protein pool. According to the shift in molecular weight of the modified A20, the main band could correspond to modification by a member of the ubiquitin family rather than phosphorylation, which will be more difficult to resolve on a 10% polyacrylamide gel. Furthermore, after CD3/CD28 stimulation we also observed multiple slow migrating forms of A20, disposed in a pattern that is more typical of polyubiquitylation (Figure 2b). However, treatment with the proteasome inhibitor MG132 did not affect the amount or the accumulation of high-molecular-weight forms of A20 (data not shown). Altogether, the different experiments presented in these figures clearly demonstrate that RNF114 specifically interacts with A20, and perhaps more specifically with a modified fraction of total A20.

### Effect of RNF114 on A20 ubiquitylation

**Figure 3:**
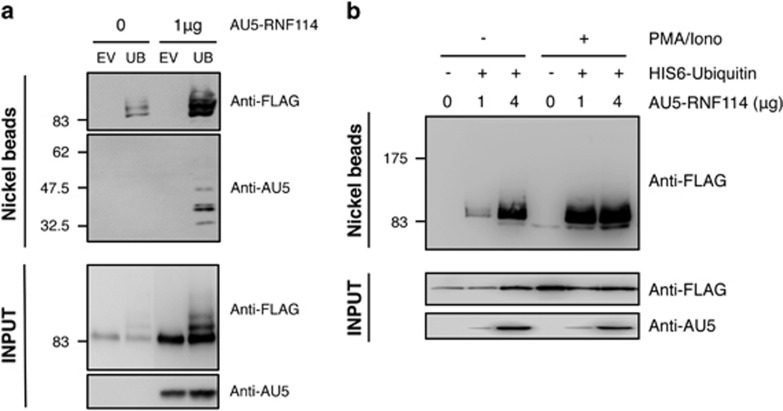
Effect of RNF114 on A20 ubiquitylation. (**a**) HEK293 cells were transfected with FLAG-A20WT in the presence or not of His6-ubiquitin and AU5-RNF114 as indicated. His_6_-ubiquitylated proteins were purified using denaturing conditions and Ni^2+^ chromatography. EV, empty vector; UB, His_6_-ubiquitin. (**b**) HEK293 cells were transfected with FLAG-A20WT in the presence or not of His6-ubiquitin and different amounts of AU5-RNF114 as indicated. Cells were stimulated for 30 min or not with PMA and ionomycin. His_6_-ubiquitylated proteins were purified using denaturing conditions and Ni^2+^ chromatography

RNF114 belongs to a novel family of ubiquitin ligases with zinc-fingers and an ubiquitin-binding domain, like the T-cell regulator RNF125/TRAC-1.^24, 25^ Therefore, we investigated whether RNF114 could promote A20 modification by coexpressing in HEK293 cells His6-ubiquitin and FLAG-A20 in the presence or not of AU5-RNF114. As shown in Figure 3a, A20 is modified with ubiquitin in the absence of AU5-RNF114; however, ubiquitylated A20 significantly increased when RNF114 is expressed. In addition, we can observe than RNF114 increases the expression level of A20 in the absence of His6-ubiquitin (Input, anti-FLAG; Figure 3a). Ubiquitylation of RNF114 itself can also be seen under these conditions (Figure 3a). As TCR stimulation induced a modification of the molecular weight of A20-bound to RNF114, suggesting a post-translational modification of A20 (Figure 2b), we checked whether RNF114-induced A20 ubiquitylation was increased after phorbol 12-myristate 13-acetate (PMA)/ionomycin in HEK293 cells. Our results revealed that level of ubiquitylated A20 increased in the presence of RNF114 and, interestingly, this effect is more pronounced after PMA/ionomycin treatment, considered as a ‘TCR-like' stimulus (Figure 3b). These results indicate that RNF114 promotes the ubiquitylation of A20.

### RNF114 induces the stabilization of NF-*κ*B regulators

**Figure 4:**
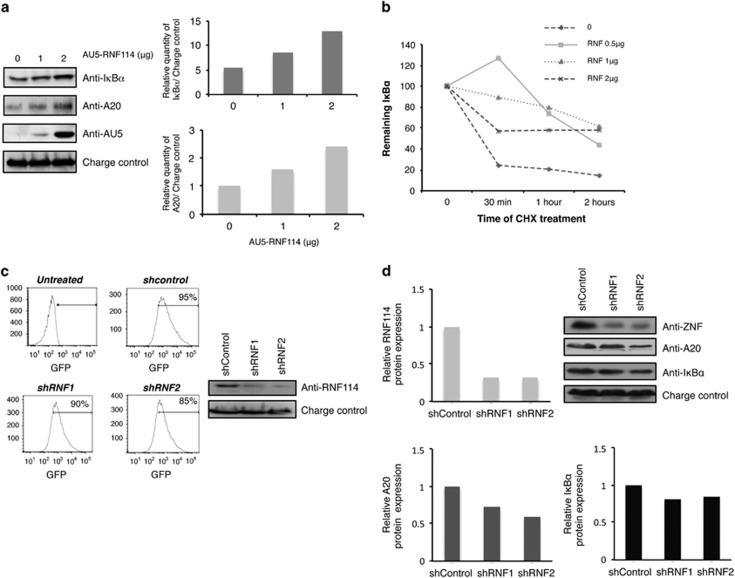
RNF114 increases the stability of both I*κ*B*α* and A20. (**a**) HEK293 cells were transfected with different amounts of AU5-RNF114. Western blot analysis with anti-I*κ*B*α*, -A20, -AU5 and -GAPDH (charge control) antibodies are presented. Graphs representing the relative amounts of I*κ*B*α* or A20 *versus* the charge control (GAPDH) of the western blots are shown. Representative result of different experiments is presented. (**b**) HEK293 cells were transfected with different amounts of RNF114 and treated for the indicated times with CHX (10 *μ*g/ml). A representative graph of three independent experiments is shown. (**c**) RNF114-specific gene silencing by lentivirus-mediated shRNA in Jurkat T cells. (Left panel) The percentage of infected cells was quantified by flow cytometry. (Right panel) Levels of protein expression were analyzed by western blot with the indicated antibodies. (**d**) Analysis of RNF114, A20 and I*κ*B*α* protein expressions in Jurkat T cells transduced with shControl or shRNF1 and 2. Western blot analysis with RNF114, A20 and I*κ*B*α* antibodies are presented as well as a corresponding quantification. Representative result of different experiments is presented

To explore the role of RNF114 on NF-*κ*B pathway, we evaluated the effects of RNF114 overexpression on the stability of two NF-*κ*B regulators, A20 and I*κ*B*α*. We can observe on the blot (Figure 4a, left panel) as well as on the corresponding quantification (Figure 4a, right panel) an accumulation of endogenous I*κ*B*α* and A20 levels in the presence of increasing amount of RNF114. The effect of RNF114 on I*κ*B*α* stability was also evaluated in a pulse-chase experiment in the presence of 10 μg/ml of cycloheximide (CHX) (Figure 4b). We observed that I*κ*B*α* was better stabilized when low levels of RNF114 were expressed, suggesting that other possible targets could be affected when high doses of RNF114 are used. To confirm the implication of RNF114 on A20 and I*κ*B*α* stabilities, we used a GFP-expressing lentiviral vector to transduce human Jurkat T cells with specific RNF114-shRNA. We used two different shRNA sequences against RNF114 (shRNF1 and shRNF2) to reduce possible off-target effects. The percentage of infection obtained was ∼90% for the two tested constructs, as well as for the control (Figure 4c, left panel) and the efficiency of endogenous RNF114 knockdown was confirmed by western blot (Figure 4c, right panel). When the expression of RNF114 was knocked down in Jurkat T cells, we observed a slight but consistent decrease of A20 and I*κ*B*α* expression. Corresponding western blot as well as its quantification is shown in Figure 4d. Altogether, the results presented here demonstrate that RNF114 plays a role in the regulation of I*κ*B*α* and A20 stabilities. In addition, these results suggest that RNF114-induced A20 ubiquitylation would be responsible for its stabilization rather than its degradation.

### Effect of RNF114 on NF-*κ*B-dependent transcription

**Figure 5:**
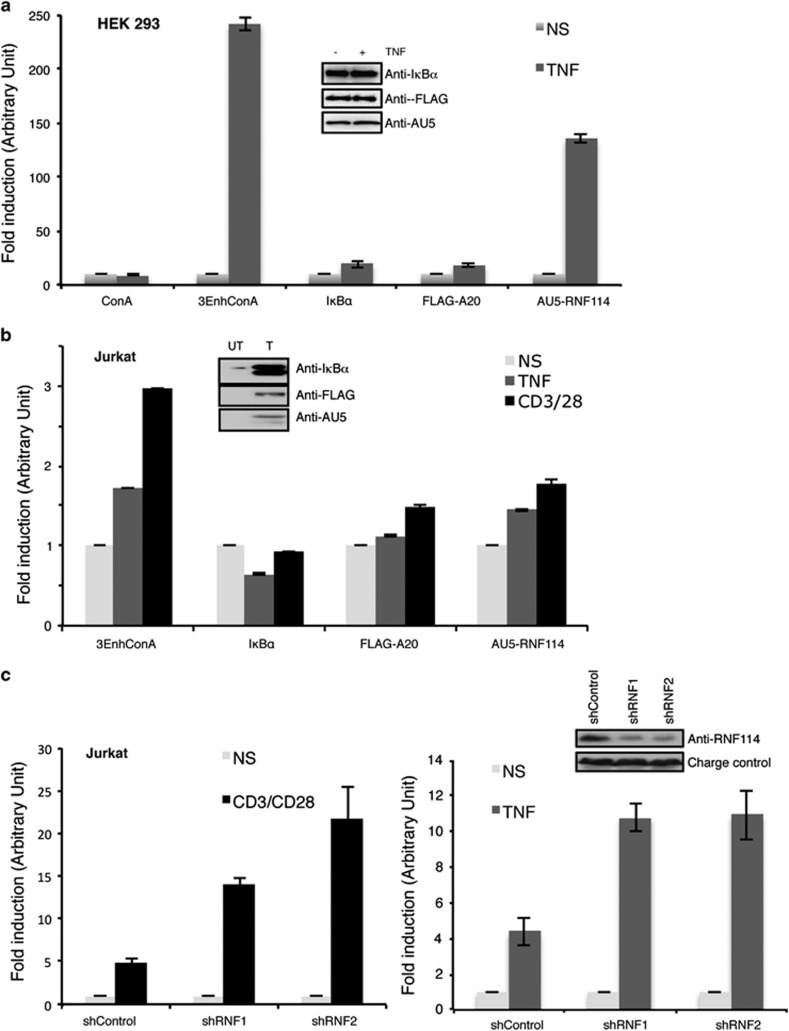
RNF114 is involved in the regulation of NF-*κ*B activity. (**a**) Luciferase assay using the 3-*κ*B enhancer ConA-luciferase plasmid (3-EnhConA) in the presence of the indicated vectors in HEK293 cells stimulated or not with TNF*α* (5 h). The graph represents the mean of three independent experiments done in triplicate. Levels of protein expression were analyzed by western blot with the indicated antibodies. (**b**) Luciferase assay using the 3-EnhConA reporter plasmids in the presence of the indicated vectors in Jurkat T cells stimulated for 5 h or not with TNF*α* or with the anti-CD3/CD28 antibodies. The graph represents the mean of three independent experiments done in triplicate. Levels of protein expression were analyzed by western blot with the indicated antibodies. (**c**) Luciferase assay using the 3-EnhConA reporter plasmids in Jurkat T cells stably transduced with shcontrol, shRNF1 or shRNF2 and stimulated or not with anti-CD3/CD28 antibodies (left panel) or with the TNF*α* (right panel). Each graph represents the mean of three independent experiments done in triplicate

Because of the important role of A20 in the regulation of NF-*κ*B, we evaluated the effects of RNF114 on the function of this transcription factor. Luciferase assays were performed in both HEK293 (Figure 5a) and Jurkat T (Figure 5b) cells using the NF-*κ*B reporter (3-*κ*B enhancer ConA-luciferase plasmid).^32^ Overexpression of RNF114 significantly attenuated TNF-induced NF-*κ*B activation in HEK293 and Jurkat T cells, as well as after TCR stimulation in Jurkat T cells (Figures 5a and b). To confirm the implication of RNF114 in the regulation of NF-*κ*B, we used the previous Jurkat T cells stably transduced with shRNF114 (Figure 4c). As can be observed in Figure 5c, knockdown of endogenous RNF114 enhanced TCR- as well as TNF*α*-induced NF-*κ*B activation in Jurkat T cells. These results confirmed that RNF114 is a negative modulator of NF-*κ*B transcription pathway acting through the stabilization of A20 and I*κ*B*α* inhibitors. The role of RNF114 might be important in a cellular context or situation where alternatives to moderate NF-*κ*B pathway could be required.

### RNF114 is a regulator of TCR signaling

**Figure 6:**
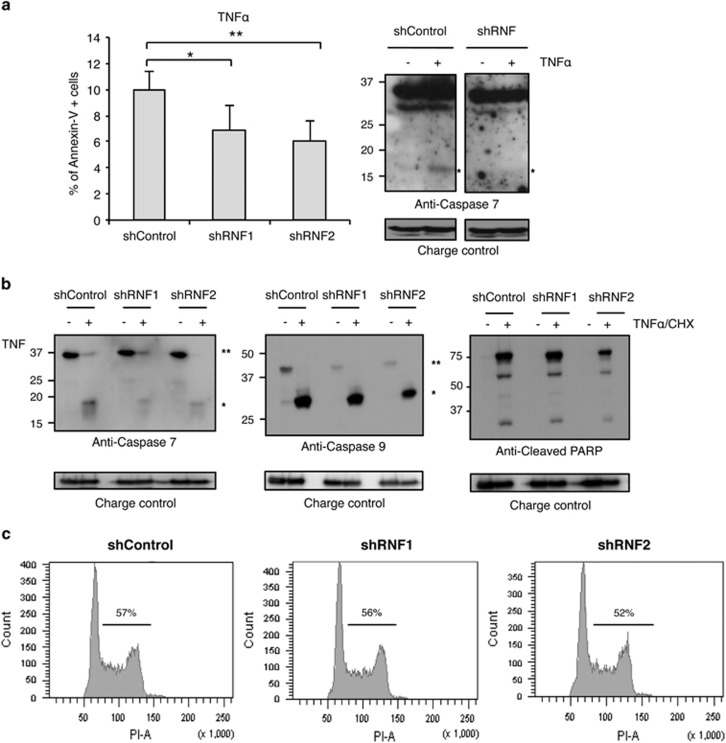
RNF114 does not modulate apoptosis or cell cycle in T cells. (**a**) Jurkat T cells stably transduced with shControl, shRNF114-1 or shRNF114-2 were treated overnight with TNF*α* (15 ng/ml). Apoptosis was analyzed by FACS after Annexin V and 7AAD staining (the percentage of Annexin V-positive cells is shown) as well as by western blot analysis looking at the cleavage of caspase 7 induced after TNF*α* treatment (indicated by an asterisk * on the western blot). Data are expressed as the mean of three independent experiments *P<0.05, **P<0.01. (**b**) Jurkat T cells stably transduced with shControl, shRNF114-1 or shRNF114-2 were treated 6 h with a TNF*α* and CHX cocktail. Protein levels were analyzed by western blot with the indicated antibodies (*indicates cleaved form and ** indicates uncleaved form). (**c**) Cell cycle analysis of Jurkat T cells transduced with shControl, shRNF114-1 or shRNF114-2 using propidium iodide staining and FACS analysis is shown

**Figure 7:**
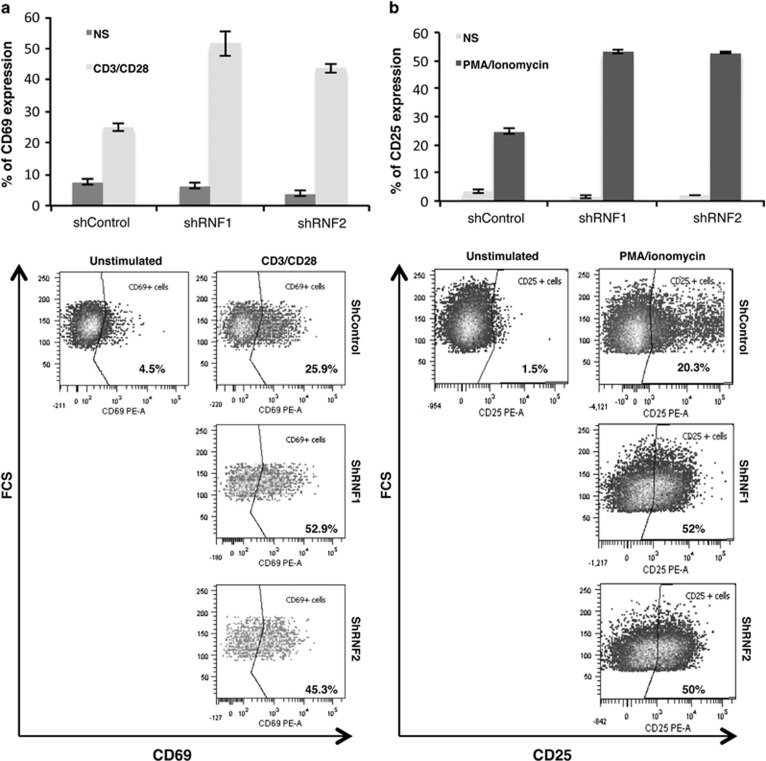
RNF114 is a regulator of TCR signaling. (**a**) Percentage of CD69-PE, obtained after FACS analysis, in Jurkat T cells stably transduced with shControl, shRNF114-1 or shRNF114-2 and stimulated or not overnight with CD3 and CD28 antibodies. A representative flow cytometry analysis of CD69 expression is shown. Background fluorescence values were set by use of isotype-matched irrelevant antibodies. (**b**) Percentage of CD25-PE, obtained after FACS analysis, in Jurkat T cells stably transduced with shControl, shRNF114-1 or shRNF114-2 and stimulated or not for 18 h with PMA/ionomycin. A representative flow cytometry analysis of CD25 expression is shown. Background fluorescence values were set by use of isotype-matched irrelevant antibodies

To determine whether RNF114 plays a role of modulator in T-cell function like its paralog TRAC1, knockdown experiments were performed using two shRNF114. First, Jurkat T cells were treated overnight with TNF*α* (15 ng/ml), known to induce T-cell apoptosis. Apoptosis events were evaluated by FACS analysis using Annexin V and 7AAD staining (Figure 6a), as well as by western blot using anti-caspase 7, caspase 9 or cleaved PARP antibodies (Figures 6a and b). When cells were treated overnight with TNF*α* alone, a reproducible inhibition of cell apoptosis was observed (Figure 6a). A mild but consistent effect is also observed on PARP or caspase-7 and -9 cleavages when cells were treated for 6 h with both TNF*α* and CHX (Figure 6b), indicating that RNF114 contributes to regulate T-cell apoptosis. However, RNF114 is not involved in the regulation of cell cycle in Jurkat T cells (Figure 6c). Then, we checked the effect of RNF114 knockdown in the regulation of T-cell activation. For that purpose, Jurkat T cells were treated overnight or not with CD3/CD28 antibodies or with PMA/ionomycin and stained with anti-CD69 and anti-CD25 antibodies (respectively) for FACS analysis. As shown in Figure 7, RNF114 knockdown induced a significant and reproducible increase of CD69 (Figure 7a) and CD25 (Figure 7b) expression, indicating that RNF114 is a negative regulator of T-cell activation.

## Discussion

The regulation of the transcription factor NF-*κ*B by posttranslational modifications with ubiquitin or ubiquitin-like proteins has been of increasing interest in recent years. NF-*κ*B not only plays a crucial role in the regulation of immune and inflammatory responses, but also ensures basic functions during cell differentiation. In this study, we identified RNF114 as a new protein interacting with the inhibitor of NF-*κ*B, A20. The domain involved in the interaction between the two proteins is present inside the E3 ligase domain of A20, suggesting that RNF114 takes part, as RNF11 or TAXBP1, in the A20 ubiquitin-editing complex. Moreover, overexpression or silencing experiments demonstrate that RNF114 is involved in the regulation of NF-*κ*B activity. However, the inhibitory function of RNF114 on the TNF*α*- or TCR-induced NF-*κ*B activation is not as strong as that mediated by A20 or I*κ*B*α* effects. The mild effects of RNF114 on NF-*κ*B activation can be explained, at least in part, by the stabilization of A20 or I*κ*B*α* inhibitors. Others studies seem to highlight that RNF114 overexpression could have an activating effect on NF-*κ*B activity.^33^ This apparent discrepancy with our results might be because of experimental differences (different cell lines, stimuli, luciferase reporters) as well as different RNF114 expression levels. Indeed, we have evidences (data not shown) that the effect of RNF114 is dose dependent, indicating that regulation of its expression level or its posttranslational modification may also be important. In fact, we observed that RNF114 is also ubiquitylated (Figure 3a), SUMOylated (data not shown) and stabilized/destabilized depending on its levels of expression (Figure 4b). Therefore, we hypothesize that RNF114 activity and function might be, like for A20, tissue and stimuli dependent.

Overexpression of RNF114 increases the stability of A20 and I*κ*B*α* and could be the mechanism by which RNF114 regulates NF-*κ*B pathway. A similar mechanism has recently been shown for the protein XAF1.^28^ However, in the case of A20, RNF114 overexpression increases A20 modification with ubiquitin without causing its degradation. Interestingly, the form of A20 bound to RNF114 in T cells after TCR stimulation undergoes a mobility shift to a form with higher molecular weight, suggesting that under these conditions RNF114 can induce A20 modification and affect its activity.

Finally, we demonstrate that in T cells, RNF114 does not appear to be involved in the regulation of cell cycle, but rather in the regulation of T-cell apoptosis and activation as its knockdown induces a decrease of TNF*α*-induced cell death and a significant increase of CD69 and CD25 expressions. Taken together, the results presented here show that RNF114 is a novel A20 interacting protein that is able to fine-tune NF-*κ*B activity in T cells stimulated with TNF*α* or anti-CD3/CD28 antibodies. RNF114 induces an increase of A20 ubiquitylation that in turn modifies A20 protein half-life. In T cells, RNF114 appears to be a modulator of A20 function and of the NF-*κ*B activity required to regulate T-cell activation. Therefore, RNF114 represents a new target candidate to develop pharmaceutical strategies to control the activation of NF-*κ*B without suppressing its full capacity as a transcriptional activator. Studying the mechanisms that allow fine-tuning of this pathway represents an alternative to modulate immune and inflammatory responses without necessarily blocking other critical functions of NF-*κ*B.

## Materials and Methods

### Cell culture and cell-based assays

HEK293 cells (ATCC, Manassas, VA, USA) were grown in DMEM (Gibco, Life Technologies, Carlsbad, CA, USA) and Jurkat cells (ATCC) in RPMI (Gibco), all supplemented with 10% FBS and antibiotics. HEK293 cells were transfected using Lipofectamine (Invitrogen, Life Technologies) and Jurkat cells were transfected by electroporation (Bio-Rad, Life Sciences, Hercules, CA, USA) as previously described.^34^

To measure transcriptional activity, cells were co-transfected with a NF-*κ*B-luciferase reporter plasmid (3-*κ*B enhancer ConA-luciferase plasmid or 3-EnhConA^32^) together with plasmids expressing FLAG-A20, AU5-RNF114 or I*κ*B*α*. Luciferase activity was measured as previously described.^35^

Cells were stimulated with either 10 ng/ml of TNF*α* (R&D Systems, Minneapolis, MN, USA), with a mixture of CD3 (OKT3) and CD28 antibodies (BD Biosciences, Franklin Lakes, NJ, USA) or with PMA (20 ng/ml, SIGMA, St. Louis, MO, USA)/ionomycin (1 mM, Calbiochem, La Jolla, CA, USA) for the indicated times.

Depletion of endogenous RNF114 expression was achieved by RNA interference. The lentiviral shRNA expression plasmids were purchased from Open Biosystems (Thermo Scientific, Denver, CO, USA). Viral particles were produced as previously described by the Viral Vector Platform at Inbiomed Foundation.^36^ Jurkat T-cell transduction was carried out at a multiplicity of infection of 10 in order to achieve 100% infection. For the luciferase experiment, cells were transfected with a NF-*κ*B-luciferase reporter plasmid (3-*κ*B enhancer ConA-luciferase plasmid) and luciferase activity was measured later.^32^

### Flow cytometry

For cell cycle analysis, Jurkat cells were washed with cold PBS and fixed with 70% ethanol overnight. Cells were then washed twice with PBS and resuspended in PBS containing 5 mg/ml propidium iodide (PI) and 10 μg/ml RNase A (Sigma-Aldrich, St. Louis, MO, USA). Cell cycle analysis was performed on GFP (530/30BP)-positive and alive cells, excluding doublets.

To track T cells undergoing apoptosis, Jurkat cells were treated overnight with TNF*α* (15 ng/ml, R&D) alone or for 6 h in combination with CHX (10 μg/ml, SIGMA). Co-staining with Annexin-V-PE (BD Biosciences; 585/42BP) and 7-aminoactinomycin D (7AAD, SIGMA; 670 LP) was performed to differentiate early and late apoptosis as well as necrotic cells. The percentage of each population was analyzed by flow cytometry gated on GFP (530/30BP)-positive cells, excluding doublets.

For activation assays, T cells were cultured as described above and stimulated for 18 h with anti-CD3 and anti-CD28 antibodies or PMA/ionomycin to check respectively CD69 and CD25 expression. CD69-PE (BD Biosciences; 585/42BP) and CD25-PE (ImmunoStep, Salamanca, Spain; 585/42BP) expressions were measured by FACS, gated on GFP-positive cells, excluding doublets and dead cells.

Data represent the mean of three independent experiments done in triplicate. A total of 10^4^ events were counted for each sample. Data were collected on a FACSCanto (BD Biosciences) and were analyzed using FlowJo software (www.flowjo.com).

### Immunodetections

Western blot detections were performed with the following primary antibodies: FLAG (SIGMA), AU5 (Covance, Princetown, NJ, USA), A20 (Calbiochem), I*κ*B*α* (Cell Signaling Technology, Beverley, MA, USA), GAPDH (SIGMA) and ZNF313/RNF114 (Santa Cruz Biotechnology, Santa Cruz, CA, USA), Sam68 (Santa Cruz Biotechnology), caspase 7 (Cell Signaling Technology), Cleaved-PARP (Cell Signaling Technology) and Caspase 9 (Cell Signaling Technology).

Co-immunoprecipitation experiments were performed using Protein-G cross-linked with 4 *μ*g of antibody/point of FLAG, AU5, A20 or RNF114 antibodies to immunoprecipitate exogenous or endogenous proteins as indicated. In all cases, cells were lysed for 15 min on ice in 50 mM sodium fluoride, 5 mM tetra-sodium pyrophosphate, 10 mM *β*-glyceropyrophosphate, 1% Igepal CA-630, 2 mM EDTA, 20 mM Na_2_HPO_4_, 20 mM NaH_2_PO_4_ and 1.2 mg/ml Complete protease inhibitor cocktail (Roche, Indianapolis, IN, USA). His_6_-ubiquitylated or SUMOylated proteins were purified using denaturing conditions and Ni^2+^ chromatography as previously described.^37^

### Cloning

A plasmid encoding human ZNF313/RNF114 cDNA (RZPD) and PCR-subcloning was performed into pGEX6 and AU5 plasmids. The A20 gene was amplified from cDNA of purified T cells using the following primers 5′-ACAAACGAATTCATGGCTGAAGTCCTTC-3′ and 5′-GCCGAGGAATTCTTAGGGGCAGTTGGGCGTTTC-3′ and cloned into a pCEFL FLAG vector. Primer sequences for deletion constructs are available upon request. The accuracy of all cloning and mutagenesis procedures was verified by sequencing.

All experiments presented in this manuscript were done at least in triplicate. For luciferase experiments and FACS analysis, data represent the mean of at least three independent experiments done in triplicate.

## Abbreviations

RNF: RING finger protein
RING: really interesting new gene
ZNF: zinc-finger protein
GWAS: genome-wide association study
SNP: single-nucleotide polymorphism
TNF: tumor necrosis factor
TCR: T-cell receptor
UIM: ubiquitin-interacting motif
